# Supplementary material for: Trachemys scripta Eggs as Part of a Potential In Vivo Model for Studying Sea Turtle Egg Fusariosis
Source: J Fungi (Basel). 2025 Jan 1;11(1):23. doi: 10.3390/jof11010023 (PMC11766952; doi:10.3390/jof11010023)
Supplement: Supplementary file 1 [file jof-11-00023-s001.zip › jof-3361591-supplementary.pdf]

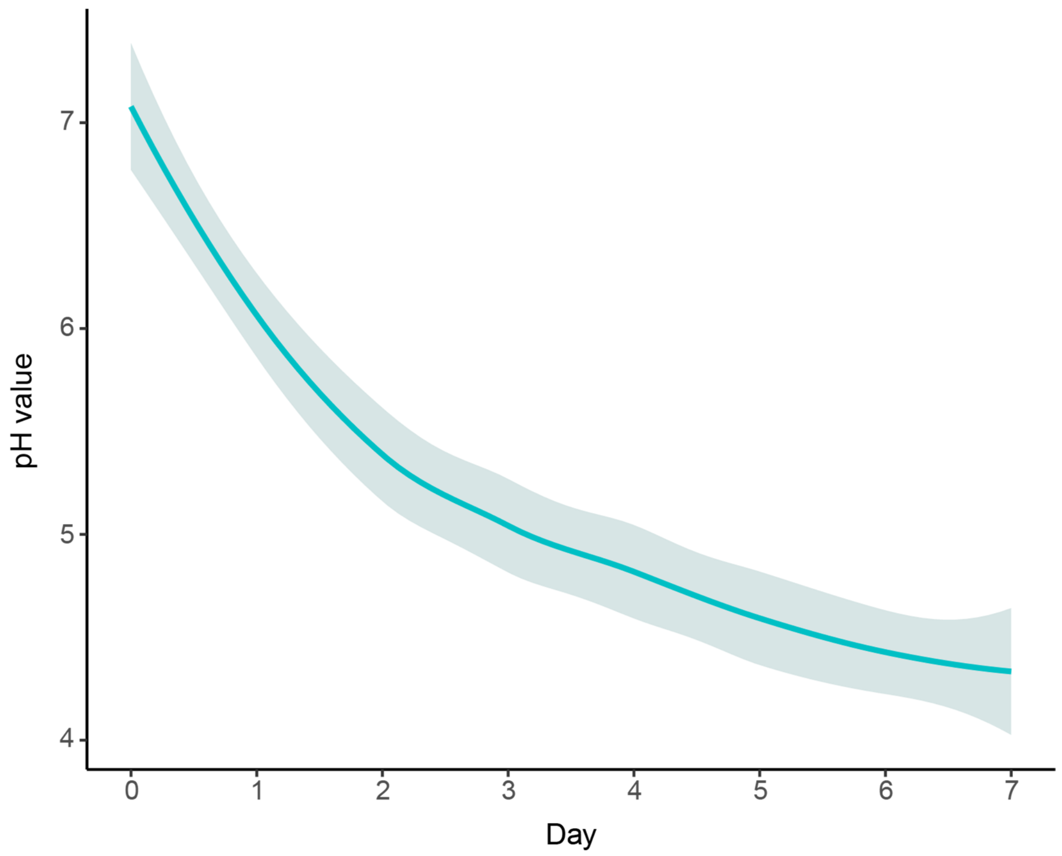

Figure S1. Acidification induced by *Fusarium keratoplasticum* isolates cultured on peptone glucose medium (initial pH = 7). The regression line was derived from the mean values of triplicate measurements taken daily. The shaded gray region represents the standard deviation (SD). No statistically significant differences in pH changes were observed among isolates ($p > 0.05$).
